# A framework of myocardial bridge detection with x-ray angiography sequence

**DOI:** 10.1186/s12938-023-01163-2

**Published:** 2023-10-19

**Authors:** Peng Zhou, Guangpu Wang, Shuo Wang, Huanming Li, Chong Liu, Jinglai Sun, Hui Yu

**Affiliations:** 1 The School of Precision Instrument and Opto-Electronics Engineering, Tianjin University, Nankai District, No.92 Weijin Road, Tianjin, China; 2 Joint Laboratory of Intelligent Medicine, Tianjin 4Th Centre Hospital, Tianjin, China; 3 The Academy of Medical Engineering and Translational Medicine, Tianjin University, Tianjin, China

**Keywords:** Myocardial bridge detection, Coronary vessel segmentation, Vessel match and sequence information fusion, X-ray angiography

## Abstract

**Background:**

Myocardial bridges are congenital anatomical abnormalities in which myocardium covers a segment of coronary arteries, leading to stenocardia, myocardial ischemia, and sudden cardiac death in severe cases. However, automatic diagnosis of myocardial bridge presents significant challenges.

**Method:**

A novel framework of myocardial bridge detection with x-ray angiography sequence is proposed, which can realize automatic detection of vessel stenosis and myocardial bridge. Firstly, we employ a novel neural network model for coronary vessel segmentation, which consists of both CNNs and transformer structures to effectively extract both local and global information of the vessels. Secondly, we describe the vessel segment information, establish the vessel tree in the image, and fuse the vessel tree information between sequences. Finally, based on vessel stenosis detection, we realize automatic detection of the myocardial bridge by querying the blood vessels between the image sequence information.

**Results:**

In experiment, we evaluate the segmentation results using two metrics, Dice and ASD, and achieve scores of 0.917 and 1.39, respectively. In the stenosis detection, we achieve an average accuracy rate of 92.7% in stenosis detection among 262 stenoses. In multi-frame image processing, vessels in different frames can be well-matched, and the accuracy of myocardial bridge detection achieves 75%.

**Conclusions:**

Our experimental results demonstrate that the algorithm can automatically detect stenosis and myocardial bridge, providing a new idea for subsequent automatic diagnosis of coronary vessels.

## Background

Coronary artery disease (CAD) has the characteristics of high morbidity and high mortality and has been a major cause of mortality worldwide and a serious threat to human health [1]. Myocardial bridge is an important disease in CAD, which is a congenital anatomical abnormality that is characterized by myocardium covering a segment of coronary arteries [2]. Myocardial bridge can be formed anywhere on the epicardial artery, but most of them occur in the left anterior descending branch [2]. Although myocardial bridge is a normal variant and generally appears to be harmless, patients with myocardial bridge can present with symptoms, such as stenocardia, arrhythmia, myocardial ischemia, and sudden cardiac death [3]. In clinical, conventional coronary angiography (CCA) has always been the gold standard for CAD diagnosis, especially for myocardial bridge [4], relying on the physician’s observation and subjective judgment to obtain the diagnosis result. On CCA, a significant “milking effect” is present at the myocardial bridge, when there is a more than 70% reduction in minimal luminal diameter during systole and a persistent more than 35% reduction in minimal luminal diameter during mid-to-late diastole [5]. Whether these conditions are met or not is judged by physicians, hence, there must exist subjectivity.

**Fig. 1:**
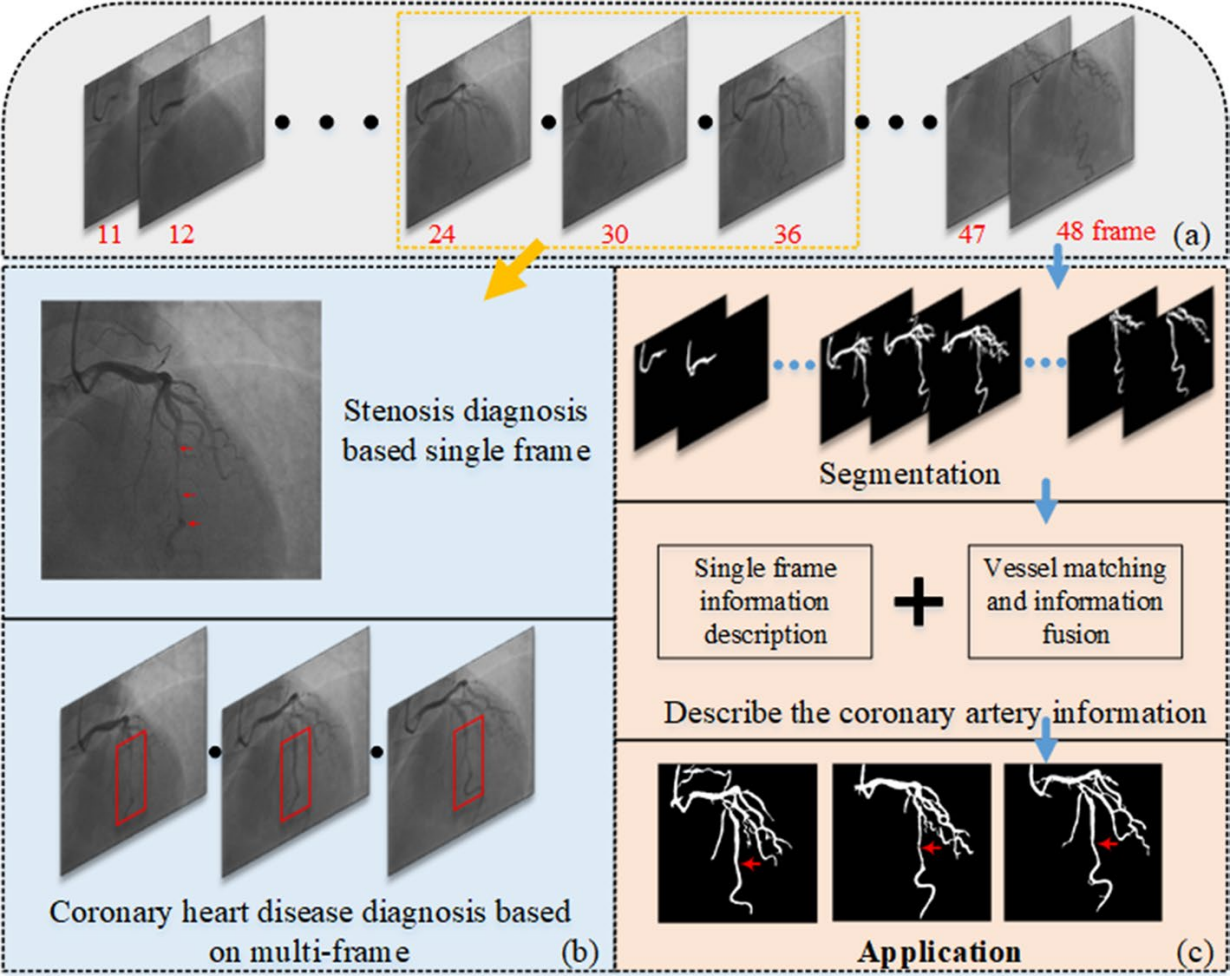
The process of coronary heart disease diagnosis: **a** an example of a coronary angiography sequence; **b** an example of a physician diagnostic process; **c** the simple workflow of this paper

CCA requires a high level of physician expertise, combining anatomical knowledge and rich clinical experience to rapidly obtain diagnostic results. A coronary angiography sequence is shown in Fig. 1a, in which the entire process lasted approximately 5 s. During this period, as illustrated in Fig. 1b, physicians need to quickly find key-frame to diagnose stenosis, and quickly obtain and compare multi-frame information to diagnose other diseases. Moreover, there is great individual variability in the coronary vessels, so physicians need to combine knowledge of coronary anatomy and clinical experience in the diagnostic process. The automatic diagnosis of CAD, especially for myocardial bridges, poses great challenges. Firstly, the coronary angiography image quality is poor and there is interference from other organs in the image, such as the lung, heart, spine, and ribs. Secondly, coronary angiography is a projection of vessels from 3 to 2 dimensions, resulting in vessel overlap, which is indistinguishable. In clinical, physicians can observe through different positions, focusing on only specific vessels in one position. For example, in the position shown in Fig. 1a, physicians focus only on the vessels that do not overlap in the lower left part of the image. Finally, it is difficult to extract coronary multi-frame information and utilize coronary anatomical information. That is the key to diagnosing CAD, especially myocardial bridge, relies on identifying periodic vessel stenosis on coronary angiography. Hence, myocardial bridges are detected by detecting whether there is periodic stenosis of vessels on coronary angiography.

In this paper, a novel framework of myocardial bridge detection with x-ray angiography sequence is proposed, which enables automatic detection of vessel stenosis and myocardial bridge. Firstly, we employ a novel neural network model for coronary vessel segmentation, which consists of both CNNs and transformer structures to effectively extract both local and global information of the vessels. Secondly, we utilize the Zhang-Suen algorithm to obtain the vessel skeleton map. We then combine the coronary segmentation image and the skeleton map to calculate the vessel diameter, length, and other relevant information. Then, we establish the vessel tree in the image and fuse the information of the vessel tree between the sequences. Finally, based on vessel stenosis detection, automatic detection of the myocardial bridge is realized by querying the blood vessels between the image sequence information. The workflow is shown in Fig. 1c.

This work has the following contributions. First, we have achieved the automatic detection of myocardial bridge. Second, we have proposed a coronary tree sequence information fusion method that can capture more angiographic information and align with the clinical diagnosis mode. Third, we have used a novel neural network model to segment coronary vessels that combines CNNs and transformer structures, and leverages the strengths of both CNNs and transformers. Fourth, we have provided a new idea for the subsequent automatic auxiliary diagnosis of CAD, and more diseases can be detected by integrating more image information.

## Related works

Coronary vessel segmentation is an important step in this paper. However, with the complex vessel structure, image noise, poor contrast, and non-uniform illumination appearing in angiograms, there are huge challenges in realizing segmentation of a coronary vessel. Coronary vessel segmentation is actually the detection and extraction of coronary blood vessels. Currently, the commonly used method of coronary vessel detection is to obtain vessel information at different scales and directions through the Hessian multi-scale enhancement method [6, 7]. And then on this basis, Kerkeni et al. and Wan et al. proposed a region growing method based on directional information and a statistical region merging to segment blood vessels separately [8, 9]. The drawbacks of the Hessian multi-scale enhancement approaches are that they are highly sensitive to noise due to the second-order derivatives. The enhancement result of small scale vessels with low contrast against the background is awful. In addition, this approach utilizes the anisotropy of the vascular structure to extract blood vessels, but it has strong isotropy at the large curvature and the intersection of the blood vessels so that the enhancement result in these parts is also awful. To overcome the above problems, Li et al. presented a robust coronary artery identification and centerline extraction method in angiographies [10]. First, the rough segmentation of the blood vessel was obtained by threshold segmentation, then the centerline of the blood vessel was extracted, and the segmentation results were further patched through the centerline. However, this requires larger calculations. Coronary vessel segmentation by convolutional neural networks (CNNs) is yet limited, because the precise annotation of a coronary vessel is extremely labor-intensive. Therefore, Zhang et al. presented weakly supervised vessel segmentation in x-ray angiograms by self-paced learning from noisy labels with suggestive annotation [11]. Which is semi-automatic and requires manual intervention. Liang et al. presented a semi 3D architecture model to segment coronary vessel from angiography, which realized the automatic segmentation of vessels. However, this method ignores the global information of vessels, due to the shortcomings of CNNs [12].

Calculating the coronary vessel information is the main content of this paper. We establish a vessel tree which is a spatially located tripartite tree and contains all the vessels in the image. Each vessel segment contains information about diameter, length, start and end position coordinates. However, the current research mainly utilizes the diameter information of the vessel to achieve the detection of stenosis. Hereafter, based on the vessel tree in the image, the fore and aft frame images are matched and correlated, which is the basis of the downstream task. Image matching can be divided into gray-based matching methods and feature-based matching methods. Due to the various noise in coronary angiography, it is not suitable for gray-based matching methods in this scene. The classic feature-based matching methods have Scale Invariant Feature Transform (SIFT) [13, 14] and Speed Up Robust Feature (SURF) et al. [15]. With reference to those methods, we utilize the vessel segment information to achieve vessel matching.

Myocardial bridges are detected by detecting periodic stenosis of vessels on coronary angiography. Currently, research on coronary vessel stenosis is the most common. At the earliest, coronary vessel stenosis was assessed by computed tomography angiography [16–19]. Subsequently, Janssen et al. proposed a method for evaluating XCA artery diameter and reference diameter based on bifurcation analysis [20]. Rfm et al. detected narrowed coronary arteries in X-ray angiographic images based on Hessian filter and wavelet fusion images [21]. Brieva et al. developed a coronary extraction and stenosis quantification method in XCA using a deformable spline algorithm and string matching technology [22]. It is worth noting that these methods are not fully automatic and require human interaction. Although Wan et al. presented a method based on CCA to automatically quantify and evaluate coronary vessel stenosis [23], the simple vessel segmentation algorithm requires many extra calculations to obtain accurate vessel centerlines and vessel diameters. With the application of artificial intelligence in the field of medical imaging, Zhang et al. proposed multi-view learning using the self-attention mechanism to directly quantify coronary artery stenosis [24]. Cong et al. proposed a model combining Inception v3 and LSTM to realize automatic detection and classification of coronary stenosis [25]. In addition, Jhm et al., Wu et al. and Ovalle-Magallanes et al. presented a CNNs model for vessel stenosis detection respectively [26–28]. These methods share a similar process. They first select a key-frame using a model, then perform vessel stenosis detection. They also skip the detection and extraction of blood vessels. Instead, they use the model to automatically detect stenosis and evaluate the degree of vessel stenosis with a regression model. Chen et al. proposed a new approach to detecting stenosis, which involved first segmenting the vessels and then detecting the stenosis [29]. Notice, all current methods were aimed at the detection of stenosis in a single image. As we know, CCA is a sequential angiography, which records the process of the contrast agent flowing with the blood over a period of time and reflects the status of the current vessel. A single angiography image cannot contain all the angiography information, and the diagnosis of some diseases in CAD also requires reference to the information of multiple frames of adjacent images. Myocardial bridge is one such disease. However, there is no research on the automatic detection of myocardial bridges based on multi-frame images with X-ray angiography. Therefore, the work of this paper is of great significance.

## Results

### Data description

Coronary angiography data were collected from Tianjin 4TH Centre Hospital from 2010 to 2018. All cases are implemented following standard guidelines and rules. All cases are anonymized. A total of 80 patients were obtained. For angiography, the device settings are the same. The frame per second is 15, and the X-Ray tube current is 56.9 kVp, and the X-Ray tube current is 92 mA. The image resolution is 512*512. To ensure the quality of the reference label, the data have been carefully studied and marked by experienced experts. The information in the image (such as patient information and hospital information, etc.) has been removed when marked by experts. In all datasets, 2661 images had segmentation labels, which all were annotated manually by physicians. Since myocardial bridges are uncommon, there were 9 real and 11 synthetic myocardial bridges among the 20 cases. The synthetic myocardial bridges are artificial to simulate myocardial bridge symptoms in cases which are physician-selected and contain non-obvious myocardial bridge symptoms. We found 262 stenoses in 23 sequences, including 66 cases of mild stenosis, 103 cases of moderate stenosis, and 93 cases of severe stenosis.

### Segmentation results and evaluation

We use two evaluation indicators, namely, the Dice and Average Symmetric Surface Distance (ASD). The Dice coefficient is a measure of the overlapping area, defined as:

1 $${\text{Dice}} = \frac{{2\left( {\Omega_{{\text{mc}}} \cap \Omega_{{\text{seg}}} } \right)}}{{\Omega_{{\text{mc}}} \cup \Omega_{{\text{seg}}} }} \times 100\%$$

where $\Omega_{{\text{mc}}}$ is the real area drawn under the guidance of the physician, and $\Omega_{{\text{seg}}}$ represents the automatic segmentation area. Dice is always between 0 and 1. The higher the coefficient, the higher the overlap between automatic and manual segmentation. ASD is defined as:

2 $${\text{ASD = }}\frac{1}{{\left| {S\left( A \right)} \right| + S\left( B \right)}}\left( {\sum_{a \in S\left( A \right)} {{\mathop {\min }\limits_{b \in S\left( B \right)}} \left\| {a - b} \right\| + \sum_{b \in S\left( B \right)} {{\mathop {\min }\limits_{a \in S\left( A \right)}} \left\| {b - a} \right\|} } } \right)$$

where $S\left( A \right)$ and $S\left( B \right)$ denote ground truth and segmentation result, respectively. The smaller the value, the better the segmentation result.

**Fig. 2:**
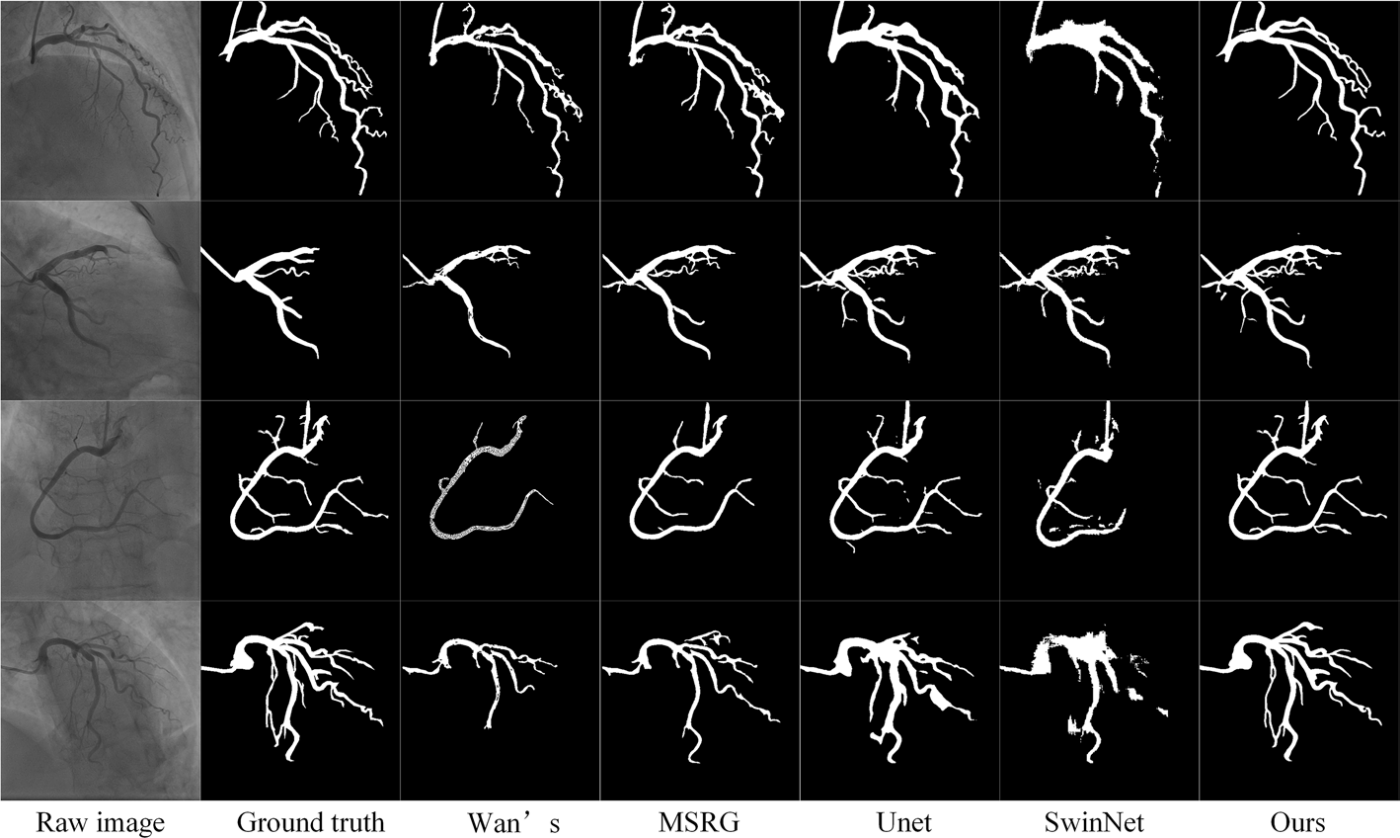
Segmentation results of three representative vessels: The first column is the raw image of the coronary angiogram. The second column is the ground truth. The third column is the segmentation result of Wan’s method. The fourth column is the segmentation result of the MSRG method. The fifth column is the segmentation result of Unet. The sixth column is the segmentation result of SwinNet. The seventh is the segmentation result of our method.

To visually show the performance of the segmentation algorithm, we have compared it with traditional methods and deep learning methods. Traditional methods include Wan’s method [22] and Kerkeni’s multiscale region growing (MSRG) method [10], respectively. Deep learning methods include Unet [30] and SwinNet [31] respectively. Figure 2 shows the segmentation results of coronary vessels. The first column is the raw image of the coronary angiography. The second column is the ground truth. The other columns are Wan’s, MSRG, Unet, SwinNet and our method, respectively. From Fig. 2, the segmentation results of deep learning method are better than traditional methods overall. In more detail, Wan’s method is the worst, it can only segment the main blood vessels, but the small blood vessels cannot be segmented at all, and there are still many holes in the segmentation results. MSRG method has achieved very good segmentation results. Not only can the main vessels be segmented well, but some small vessels can also be well-identified. Unet’s segmentation results are similar to those of MSRG. SwinNet’s results are worst among the deep learning methods, especially in the segmentation results at dense vessels. The possible reason is that the network’s own structure transformer only focuses on global information, but can’t extract local edge information. The segmentation results of our method are the best. The reason is that the network combines the advantages of CNNs and transformers, and is able to focus on both local and global information. Moreover, our method can well identify small vessels and even small vessels that physicians ignore.

In addition to visual comparisons, our method also uses 2 performance indicators to compare with the reference method. The comparison results are shown in Table 1. Through comparison, our method has the best performance, both the Dice and ASD are greatly improved with the other methods. The quantitative results are consistent with the qualitative results shown in Fig. 2. As the segmentation of vessels is more accurate, much computing power is saved for the subsequent calculation of vessel diameter.

**Table 1:** The segmentation performance measured by the Dice and ASD

Method	Dice	ASD
Wan’s method	0.478	17.7
MSRG Unet SwinNet	0.703 0.866 0.875	11.9 2.07 2.78
Ours	0.917	1.39

### Stenosis and myocardial bridge

Clinically, vessel stenosis is divided into three grades: mild stenosis (30% to 50%), moderate stenosis (50% to 70%), and severe stenosis (over 70%). The corresponding labels of vessel stenosis grade are shown in Table 2. All case labels were marked by physicians with rich clinical experience. We use accuracy and recall to evaluate the performance of vessel stenosis detection. Accuracy and recall rate are defined as:

3 $${\text{Accuracy}} = \frac{{\text{TP + TN}}}{{{\text{TP}} + {\text{FP}} + {\text{TN}} + {\text{FN}}}}$$

4 $${\text{Recall}} = \frac{{{\text{TP}}}}{{{\text{TP}} + {\text{FN}}}}.$$

**Table 2:** Coronary stenosis grade label

Stenosis grad	Label
Mild stenosis (30% to 50%)	0
Moderate stenosis (50% to 70%)	1
Severe stenosis (over 70%)	2

**Fig. 3:**
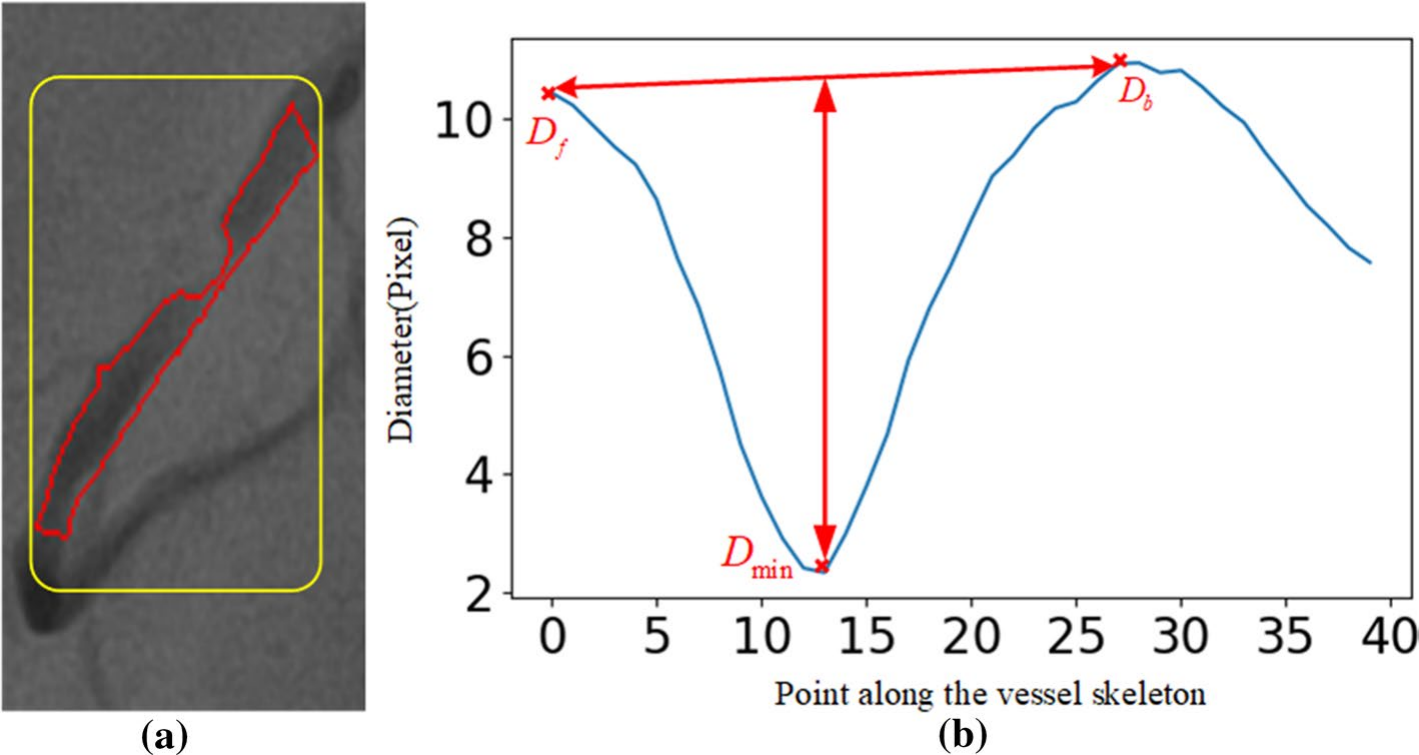
An example of detecting vessel stenosis through a vessel diameter curve. **a** is the stenotic lesion; **b** is the diameter curve at the stenotic lesion

Figure 3 shows an example of detecting vessel stenosis through a vessel diameter curve. The diameter curve in Fig. 3b shows the computed and smoothed diameter of the vessel in Fig. 3a. Since the vessel is not a peripheral vessel, the minimum diameter is $D_{{\text{min}}}$. Then $D_f$ and $D_b$ are found on both sides of $D_{{\text{min}}}$ to calculate the degree of vessel stenosis. In Fig. 3b, the degree of vessel stenosis is 79.3%, which belongs to the category of severe stenosis.

To show the performance of the vessel stenosis rating more intuitively, we used 262 cases of stenosis, including 66 cases of mild stenosis, 103 cases of moderate stenosis, and 93 cases of severe stenosis. The performance of vessel stenosis detection was measured by accuracy and recall rate. Table 3 lists the quantitative results of mild, moderate, and severe stenosis. This is consistent with the actual situation because the cases near the boundary of mild and moderate stenosis are prone to be misclassified as a higher or lower level of disease than their own, such as mild stenosis being misclassified as moderate stenosis or normal. In the experiment, 4 cases were misidentified as normal vessels. Moreover, physicians will take into account the significance of the vessel when making a diagnosis and adjust the severity or leniency of the judgment accordingly. This is also part of the reason for the low recall.

**Table 3:** The stenosis detection performance was measured by accuracy, recall rate

	Mild stenosis	Moderate stenosis	Severe stenosis
Accuracy	93.1%	90.0%	95.4%
Recall	83.3%	83.5%	97.8%

**Fig. 4:**
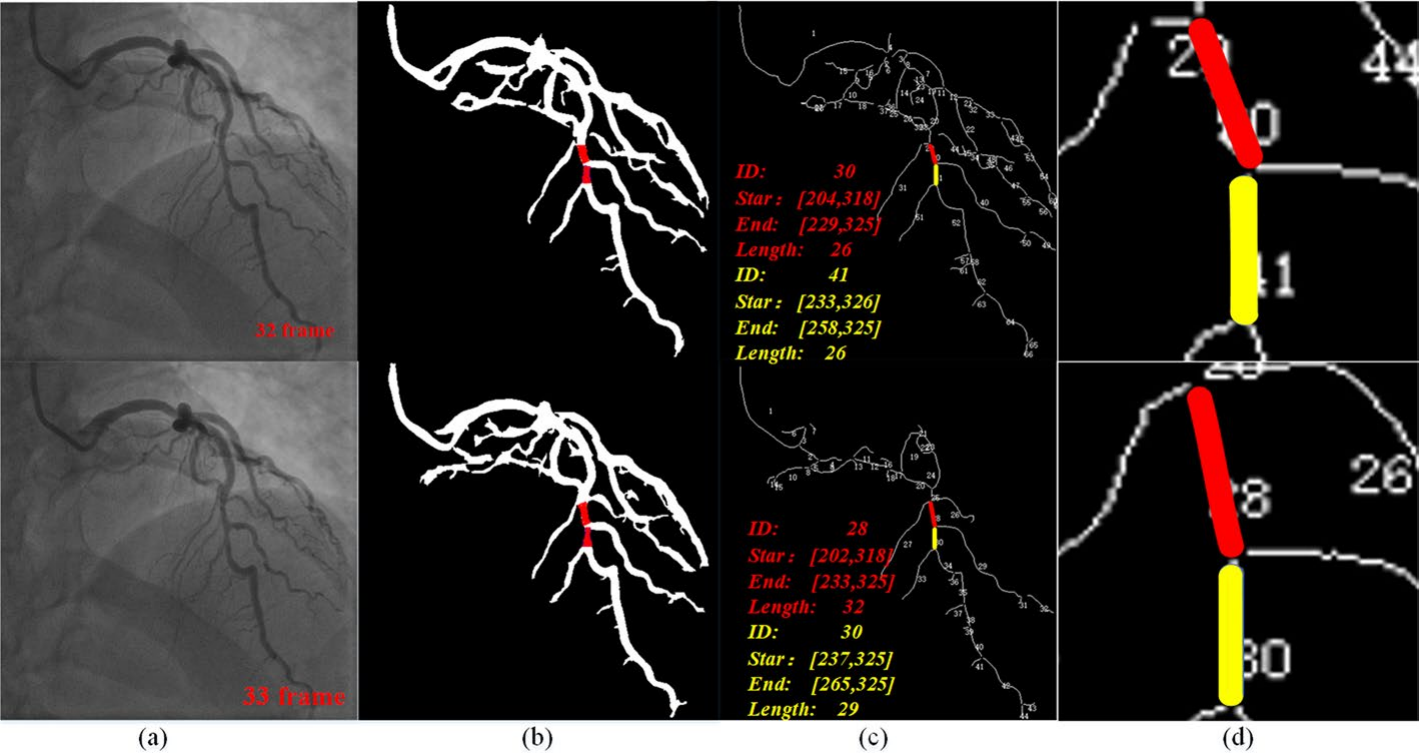
An instance of vessel matching: **a** is two adjacent original images in a coronary image; **b** is the segmentation image corresponding to the original image, in which the two pairs of matching vessels are painted in red; **c** is the corresponding vessel tree, the lower right corner is the information of the two highlighted vessels; **d** is an enlarged view of the highlighted vessels

Vessel matching is to determine the same vessel in the sequence, which is the basis of myocardial bridge detection. Figure 4 shows an instance of vessel matching: (a) is two adjacent original images in a coronary image; (b) is the segmentation image corresponding to the original image, in which the two pairs of matching vessels are painted in red; (c) is the corresponding vessel tree, the lower right corner is the information of the two highlighted vessels. (d) is an enlarged view of the highlighted vessels. As shown in Fig. 4, the vessel with ID 30 in the 32^nd^ frame of the coronary image and the vessels with ID 28 and 30 in the 33rd frame all satisfy the matching condition, because the vessels are relatively short, and the threshold of the vessel matching condition is set high for universality. If the matching is not optimized, the wrong vessel will be matched due to the sequence of matching detection. Hence, we add the optimization of the matching algorithm. When the above situation occurs, calculate the sum of $d_s$ and $d_e$ in method Eq. (12), respectively, and choose the vessel with the smallest value as the matching vessel.

**Fig. 5:**
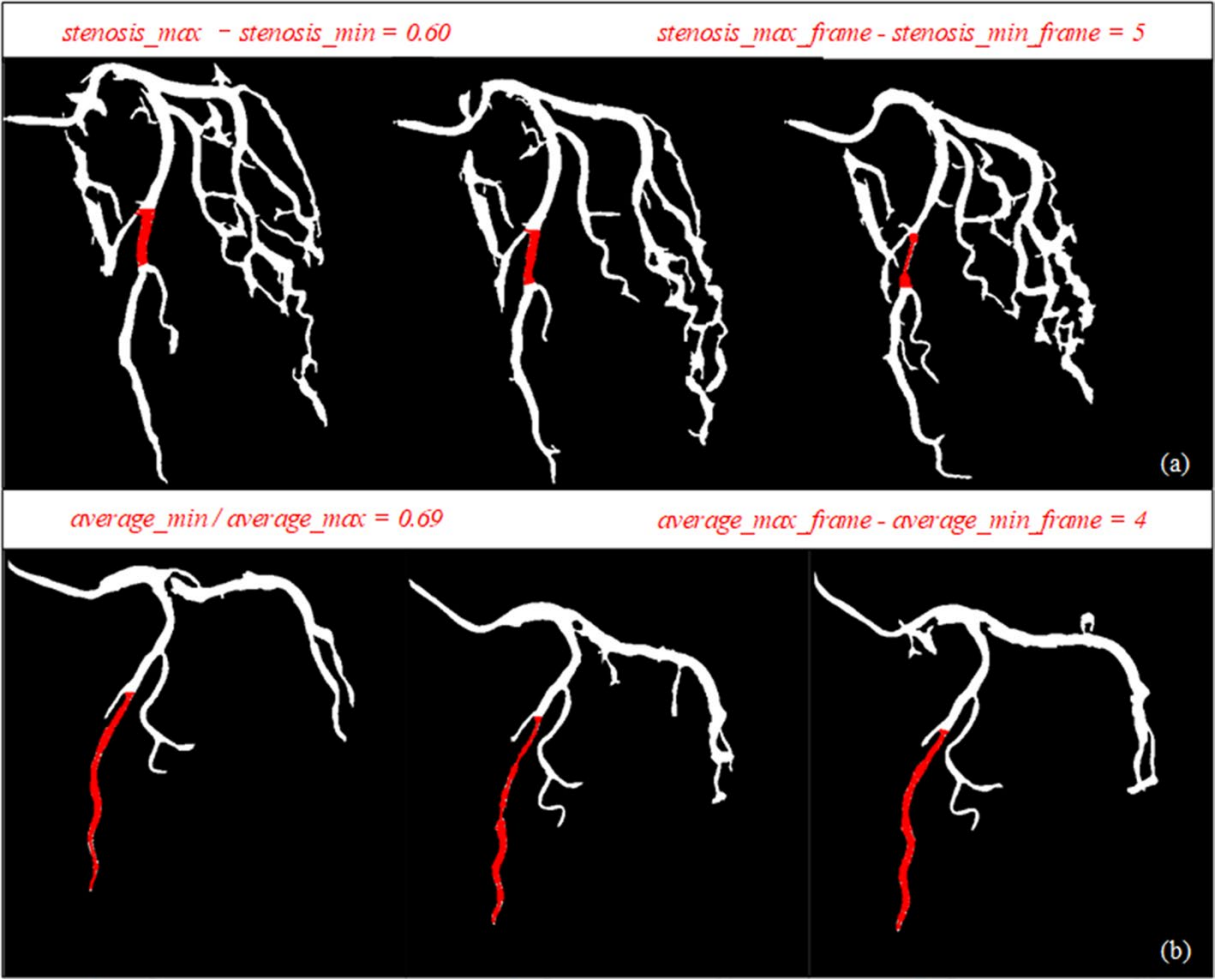
An instance of myocardial bridge detection: **a** is judged by the change in the degree of blood vessel stenosis. **b** is judged by the change in blood vessel diameter

Combining the blood vessel matching information between coronary sequences and vessel stenosis detection can realize the detection of myocardial bridge. Firstly, identify the same vessel between different sequences by querying the fusion of the sequence information. Then, calculate the stenosis of all vessels that are identified by this method. Finally, confirm the existence of myocardial bridge. Figure 5 is an instance of myocardial bridge detection. (a) is judged by the change in the degree of vessel stenosis; (b) is judged by the change in vessel diameter. In Fig. 5a, the diseased region of the myocardial bridge is short, and stenosis can be detected. Therefore, the myocardial bridge can be easily detected by the change in the degree of stenosis. In Fig. 5b, due to the long diseased region, it is impossible to accurately calculate the degree of stenosis of the vessel. Then the myocardial bridge can be easily detected by the change in diameter. Since myocardial bridges are uncommon, there were 9 real and 11 synthetic myocardial bridges among the 20 cases, the accuracy of myocardial bridge detection achieved 75%.

## Discussion

In this paper, we implemented myocardial bridge detection based on x-ray angiography. Coronary vessel segmentation is an important step in this paper. We use a new neural network model which contains CNN and transformer structures to segment coronary vessels. However, structures similar to vessels in the images, such as ribs, cannot be distinguished, resulting in incorrect segmentation. In the experiment, 20 myocardial bridge cases were used in this paper, of which 9 cases were real data and 11 cases were synthetic data. Since myocardial bridges are not a common coronary disease, case data are more difficult to obtain and synthetic data can achieve the expected experimental results over. In this paper, myocardial bridges were detected with an accuracy of 75%. The reasons for the poor accuracy are: Firstly, the coronary arteries have great individual variation and the lesions can be located in diverse places; secondly, this paper focuses on the detection of supravascular myocardial bridges and neglects the location of vessel forks; thirdly, the overlap of vessels during imaging leads to undetectability.

Currently, deep learning is becoming more and more popular and is applied in many medical fields. However, a common problem is the acquisition of high-quality dataset and label [35], especially for the coronary vessel. Moreover, coronary angiography data are stored in video form, making data annotation more challenging. Intelligent auxiliary diagnosis of CAD based on coronary angiography video will be our future work.

## Conclusions

Due to the complex structure and complex background of vessels, automatic diagnosis of coronary artery diseases, especially myocardial bridge, presents a huge challenge. In this paper, we propose coronary vessel tree sequence information fusion based on x-ray images and use coronary sequence information to realize the automatic detection of stenosis and myocardial bridge. Firstly, we use a new neural network model which contains CNN and transformer structures to segment coronary vessels, and then describe the blood vessel information from the three levels of blood vessel segments, vessel tree, and sequence. Finally, the detection of vessel stenosis and the detection of myocardial bridge are realized. We performed quantitative and qualitative performance evaluations on the segmentation results and the detection of vessel stenosis. Compared with Wan’s method, MSRG, Unet and SwinNet, the segmentation result of our method is optimal. Dice and ASD reached 0.917 and 1.39, respectively. Our stenosis detection method can accurately locate and classify the degree of stenosis, with mild, moderate, and severe stenosis accuracy rates reaching 93.1%, 90.0%, and 95.4%, respectively. In multi-frame image processing, vessels in the different frames can be well-matched, and the accuracy of myocardial bridge detection achieved 75%. Experimental results show that this method can be used for automatic detection of myocardial bridges and stenosis, and provides a new idea for the subsequent automatic assisted diagnosis of a coronary vessel.

At present, our method still has certain limitations. Since the research content of this paper relies heavily on the segmentation results of the first step, and the segmentation results of images with very complex backgrounds are poor, which leads to the subsequent results also becoming poor. In future work, we will strive to overcome these limitations. In addition, there is also the phenomenon of overlapping crossed vessels in coronary angiography, which is caused by two main reasons: the first one is the tortuosity of the vessel itself; the second one is that the two vessels projection overlap. Which is a problem we cannot overcome at this time. In future work, we will focus on overcoming these limitations by multi-body vessel matching with coronary angiography.

## Methods

**Fig. 6:**
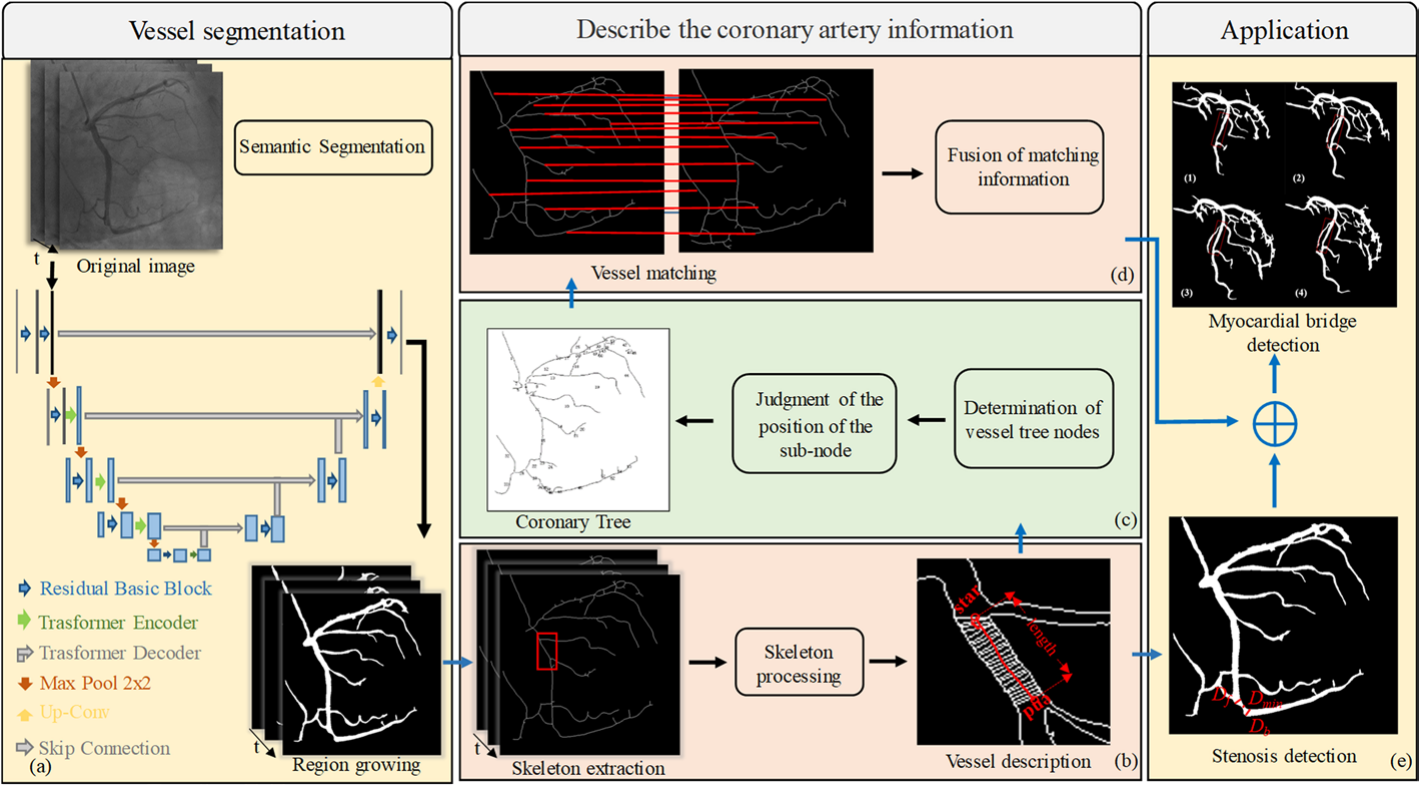
The workflow of this paper: **a** Obtain coronary segmentation images through our neural network model. **b** Extract the vessel skeleton, and describe the vessel segment information, including the diameter, length, start and end position coordinates of the blood vessel. **c** Construct a multi-way tree vessel tree based on spatial location. **d** Matching and correlation of vessels in the images of the previous and subsequent frames. **e** Detection of vessel stenosis and myocardial bridge detection

In this study, we propose a novel framework of myocardial bridge detection with x-ray angiography sequence, which can play an important role in automatic detection of vessel stenosis and myocardial bridge. As illustrated in Fig. 6, the proposed method is presented in three stages: Firstly, we use a new neural network model which uses UTNet [32] as backbone and contains CNNs and transformer structures to segment coronary vessels. Secondly, the coronary vessel information is calculated, which is divided into three steps: extract the vessel skeleton, calculate the vessel segment information, including the diameter, length, start and end position coordinates of the vessel; construct a multi-way tree vessel tree based on spatial location; match and correlate vessels in the images of the previous and subsequent frames. Finally, combining the detection of stenosis and coronary vessel information, the automatic detection of myocardial bridges is realized.

### Coronary vessel segmentation

Coronary vessel segmentation is an important part of coronary vessel auxiliary diagnosis, and the result of segmentation directly affects the follow-up work of this paper. An overview of the architecture of our model is shown in Fig. 6a. Our model is an end-to-end coronary vessel segmentation neural network. The encoder consists of residual basic block and transformer encoder. The decoder consists of residual basic block and transformer decoder. To generate a hierarchical representation, we reduce the size of the token map by max pool, which halved the map size. We have employed the transformer in both the encoder and decoder, which can capture the global information on coronary vessels. However, applying transformer too early in the very shallow layers of the network does not beneficial in experiments but introduces additional computation. Therefore, we did not introduce transformer in the first layer of the encoder and decoder. All other layers in encoder have residual basic block and transformer encoder, with transformer encoder preceding max pool. The network also uses transformer decoder instead of the regular skip connection to pass the feature layer to the decoder, except for the first layer. Notice, the transformer is a module that contains all necessary operations such as dividing the feature map into different patches and self-attention.

The transformer is built upon the multi-head self-attention (MHSA) module [33], which can jointly infer attention from different subspaces. In this study, we use 4 heads, and only the single head is shown below for simplicity. Assume an input feature map $X \in R^{C \times H \times W}$, where $H,W$ are the map size, and $C$ is the number of channels of feature map. Utilizing three $1 \times 1$ convolutions, $X$ is projected to $Q,K,V \in R^{d \times H \times W}$, where $d$ is the dimension of embedding. Then, $Q,K,V$ is flattened and transposed into sequences with size $HW \times d$. The output of the self-attention is as followed:

5 $${\text{Attention}}\left( {Q,K,V} \right) = {\text{softmax}}\left( {\frac{{QK^{\text{T}} }}{{\sqrt {d} }}} \right)V.$$

Images are highly structured data, and the pair-wise attention computation among all pixels is highly inefficient and redundant [32]. Therefore, a large amount of data are required for the network to converge, when using transformer to train network. For small sample size problem for coronary segmentation, we use small size feature map instead of high-resolution feature. The main idea is to use two small size feature maps which are obtained by sub-sampling the high-resolution features: $\overline{K},\overline{V} \in R^{k \times d}$ instead of $K,V \in R^{n \times d}$, where $K \ll n = HW$. In this paper, the feature map size we used is 16.

### Calculate the coronary vessel information

Coronary vessels are the main target of CCA and the basic unit of coronary sequence information fusion. Coronary vessel description includes the starting and ending positions, length, diameter, and ID. The premise of all work is to extract vessel skeletons, and Zhang-Suen is used to extract vessel skeletons [34]. Firstly, delete pixels which meet the following conditions:

6 $$\begin{gathered} \left\{ {\begin{array}{*{20}c} {2 \le N\left( {p1} \right) \le 6} \\ {S\left( {p1} \right) = 1} \\ {p2 \cdot p4 \cdot p6 = 0} \\ {p4 \cdot p6 \cdot p8 = 0} \\ \end{array} } \right. \hfill \\ \hfill \\ \left\{ {\begin{array}{*{20}c} {2 \le N\left( {p1} \right) \le 6} \\ {S\left( {p1} \right) = 1} \\ {p2 \cdot p4 \cdot p8 = 0} \\ {p2 \cdot p6 \cdot p8 = 0} \\ \end{array} } \right. \hfill \\ \end{gathered}$$

where $N\left( {p1} \right)$ denotes the 8-neighborhood pixels of pixel $p1$. $S\left( {p1} \right)$ denotes the number of pixel values in the 8-neighborhood pixels of pixel $p1$ from 0 to 1. Secondly, delete the bottom right and bottom left pixels of the pixel that meet Eq. 9, defined as follows:

7 $$\begin{aligned} P_x \left( {i - 1} \right) &= \left[ {P_x \left( i \right) - 1} \right]\& P_y \left( {i - 1} \right) = P_y \left( i \right)\& P_x \left( i \right) \hfill \\ & = P_x \left( {i + 1} \right)\& P_y \left( i \right) = \left[ {P_y \left( {i + 1} \right) + 1} \right] \hfill \\ P_x \left( {i - 1} \right) &= P_x \left( i \right)\& P_y \left( i \right) = \left[ {P_y \left( {i - 1} \right) + 1} \right]\& P_x \left( i \right) \hfill \\ & = \left[ {P_x \left( {i + 1} \right) - 1} \right]\& P_y \left( i \right) = P_y \left( {i + 1} \right) \hfill \\ \end{aligned}$$

where $P_x \left( i \right)$ and $P_y \left( i \right)$ denote the coordinates of pixel $p_i$, respectively.

Coronary vessel description is designed for a single vessel, so we need to separate vessels by extracting the key points of the skeleton. The key points of blood vessels include endpoints and intersections. Since the vessel skeleton is a single-pixel width, there is only one pixel in the 8-neighborhood pixels at an endpoint, while the intersection cannot rely solely on the 8-neighborhood sum of pixels to be greater than two, because the 8-neighborhood sum of the pixels near the intersection is also greater than two. Hence, we remove the false intersections based on the pixel location information in the 8 neighborhoods at the intersections. The conditions that the intersection should meet are described as follows:

8 $$\left\{ {\begin{array}{*{20}c} {N\left( {p1} \right) \ge 3} \\ {p3 + p4 + p5 \le 1} \\ {p5 + p6 + p7 \le 1} \\ {p7 + p8 + p9 \le 1} \\ {p9 + p2 + p3 \le 1} \\ \end{array} } \right.$$

where $N(p1)$ is the 8-neighborhood sum of pixel $p1$. $p2$ is directly above pixel $p1$, and other pixels are arranged clockwise.

Due to the unclear vessel edges in the coronary image, incorrect segmentation occurs during vessel segmentation, or the defect of the vessel skeleton extraction algorithm, which results in some very short vessel segments after the vessel is separated. These vessels are considered burr vessels and we need to remove them. We treat vessels with a blood vessel length of less than 6 pixels as burr vessels.

**Fig. 7:**
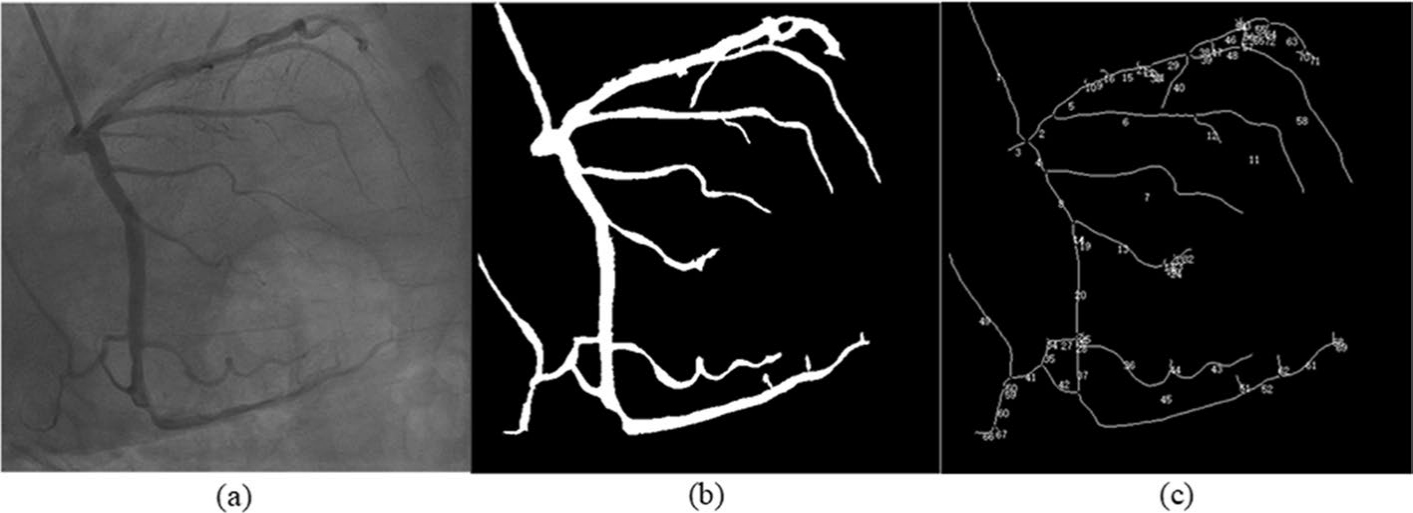
Construction of a multi-way tree vessel tree based on spatial location: **a** Original image. **b** Coronary vessel segmentation results. **c** Results of the multi-branch vessel tree

The repair of the vessel skeleton involves reconnecting a piece of vessel that was originally connected but disconnected due to the presence of burrs. The situation where vessels are separated by mistake may be caused by excessive changes in the curvature of the vessel, certain defects in the vessel skeleton extraction algorithm, etc. The original skeleton and the skeleton that has been removed burr vessels are used to repair vessel skeleton. First, the original skeleton minus the deburred skeleton obtains the deleted part as described above. And then, referring to the deleted part, trace intersections back from endpoints of the deburred skeleton. Figure 7 shows the results of skeleton repair after re-adding the pixels deleted between the head and tail of two vessels.

The diameter of a vessel is an important parameter of vessel expression, and it is also the main basis for the diagnosis of vascular disease. The diameter of a vessel is expressed by the distance between the perpendicular line of the point on the vessel skeleton and the intersection point of the vessel edge. We use the least-squares method to fit the straight line of the points on the vessel skeleton, and then use the relationship between two mutually perpendicular straight lines to find the straight line in the normal direction of the vessel. Assuming that given $N$ pixel coordinate points, the straight line can be fitted, according to the least-squares rule. The rule meets the following:

9 $$\min \left( {\sum_{i = 1}^N {\left( {ax_i + b - y_i } \right)^2 } } \right).$$

According to the value of $a$, $b$ and pixel $p_i \left( {x_i ,y_i } \right)$ on the skeleton, the skeleton normal is obtained. Finally, the coordinates of the intersection of the normal line and the edge of the vessel, $A\left( {x_a ,y_a } \right)$ and $B\left( {x_b ,y_b } \right)$, are calculated. Then, the diameter is expressed as follows:

10 $$d = \sqrt {{\left( {x_a - x_b } \right)^2 + \left( {y_a - y_b } \right)^2 }} .$$

After the above process, the start and end coordinates of the vessel are denoted by the coordinates of the two endpoints of the blood vessel skeleton, and the length is denoted by the sum of the number of pixels of the skeleton.

The coronary vessel tree integrates all the vessels in the whole picture to establish a multi-branch vessel tree based on spatial location. Coronary angiography vessels grow from top to bottom, so the root node of the tree is determined in the earliest blood vessel, that is, the catheter during angiography. The catheter usually appears in the upper left corner of the image, which is very close to the origin of the image coordinates. We utilize this feature to determine the position of the root note. By observing that there are at most three branch vessels at the vessel bifurcation, the degree of the vessel tree is set to 3. The children of a node are determined according to the distance between the endpoint of the node’s vessel and the starting point of a new vessel. Considering the amount of optimization calculation, we use the Manhattan distance definition:

11 $$d_n = \left| {x_s - x_e } \right|{ + }\left| {y_s - y_e } \right|,$$

where $x_{*} ,y_{*}$ denote pixel coordinates.

Each node has at most three sub-nodes. We determine the relative position of the sub-nodes according to the actual spatial position. The essence is to determine whether the starting point of the vessel of the sub-node and the endpoint of the vessel of the node are sorted clockwise or counterclockwise. Assuming that the node has two sub-nodes, it is to determine the sequence of the endpoint $p1\left( {x_1 ,y_1 } \right)$ of the vessel of the node and the starting points $p2\left( {x_2 ,y_2 } \right)$, $p3\left( {x_3 ,y_3 } \right)$ of the two sub-node vessels. The three points are calculated as follows:

12 $$V = \left( {x_2 - x_1 } \right) \times \left( {y_3 - y_2 } \right) - \left( {y_2 - y1} \right) \times \left( {x_3 - x_2 } \right),$$

where $V$ is greater than zero, points $p1$, $p2$ and $p3$ are arranged counterclockwise, and $p2$ is the first sub-node; otherwise, the three points are arranged clockwise, and $p3$ are the first sub-node. When there are three sub-nodes, let the endpoint of the vessel of the node and the starting point of the vessel of the sub-node perform the calculation of Eq. (9) in pairs, and then determine the arrangement of the four points to determine the first sub-node. According to the growth characteristics of coronary angiography vessels, the vessel ID value shown in Fig. 7 is assigned from top to bottom. The smaller the ID value, the earlier the blood vessel will be developed in coronary angiography.

Coronary sequence information fusion contains vessel matching and correlation of vessels in the fore large changes due to the heart beat, the topological characteristics of the vessels will not change much, such as the branch point and the length of the blood vessel, so we use starting and ending points of the blood vessel and the length of the blood vessel has been extracted as the feature of vessel matching. Assume that the starting coordinates of the two segments of coronary vessels in the previous and subsequent frames are $S_1 \left( {x_{s1} ,y_{s1} } \right)$ and $S_2 \left( {x_{s2} ,y_{s2} } \right)$, the ending coordinates are $E_1 \left( {x_{e1} ,y_{e1} } \right)$ and $E_2 \left( {x_{e2} ,y_{e2} } \right)$ , respectively, and the lengths of the two segments are $L_1$ and $L_2$ , respectively. The distances $d_s$ and $d_e$ between the start point and the endpoint of the two blood vessels are:

13 $$\begin{gathered} d_s = \left| {x_{s1} - x_{s2} } \right| + \left| {y_{s1} - y_{s2} } \right| \hfill \\ d_e = \left| {x_{e1} - x_{e2} } \right| + \left| {y_{e1} - y_{e2} } \right| \hfill \\ \end{gathered}$$

The ratio of the length of the two vessels is:

14 $${\text{Rate}} = \frac{{\min \left( {L_1 ,L_2 } \right)}}{{\max \left( {L_{1,} L_2 } \right)}}.$$

After testing and optimization, the condition for judging that two segments of vessels in two images are the same blood vessel is:

15 $$\left\{ {\begin{array}{*{20}c} {d_s < 65} \\ {d_e < 65} \\ {{\text{rate}} > 0.80} \\ \end{array} } \right.$$

To enhance the robustness of the vessel matching algorithm, in addition to matching the vessels in the neighboring images, we will also match the vessels in the neighboring second and third images. When a blood vessel has been matched, it will not be matched in subsequent images.

To quickly find and query the same vessel in the sequence, we add a new attribute to the description of the vessel, which contains the location of the vessels on coronary sequence and the images, and also records the location of the matching vessels.

### Application

Coronary vessel stenosis is the most common disease in CAD. After measuring the vessel diameter, the local extremum of the curve formed by the vessel diameters can be used to determine the location and degree of the vessel stenosis. Firstly, using filter defined as Eq. (13), we smooth the diameter curve to eliminate high-frequency noise on the curve and make the curve smoother. We use an estimation method based on the expected healthy diameter of the stenosis to quantify the degree of vessel stenosis, which also fits the physicians' diagnostic method. We regard the local minimum as the stenosis candidate position, and the average value of the local maximum on both sides as the expected diameter. The formula could be given as:

16 $$y\left( k \right) = \frac{1}{N_w }\sum_{i = 0}^{N - 1} {x\left( {k - i} \right)}$$

where $y(k)$ is the result of smooth filter, $x(k)$ is the curve before filtering. $N_w$ is the filter window length.

17 $$P = \left( {1 - \frac{{2D_{\min } }}{D_f + D_b }} \right)*100\%$$

where $P$ is the quantitative value of the degree of vessel stenosis, $D_{\min }$ is the local minimum, $D_f$ and $D_b$ are the local maximums on both sides of $D_{\min }$, respectively. Figure 3 shows the relationship between $D_{\min }$, $D_f$ and $D_b$. Before stenosis detection, the coronary vessels have been decomposed into vessel segments, so if they are in a non-peripheral vessel, then the minimum value of the whole vessel diameter is $D_{\min }$.

**Fig. 8:**
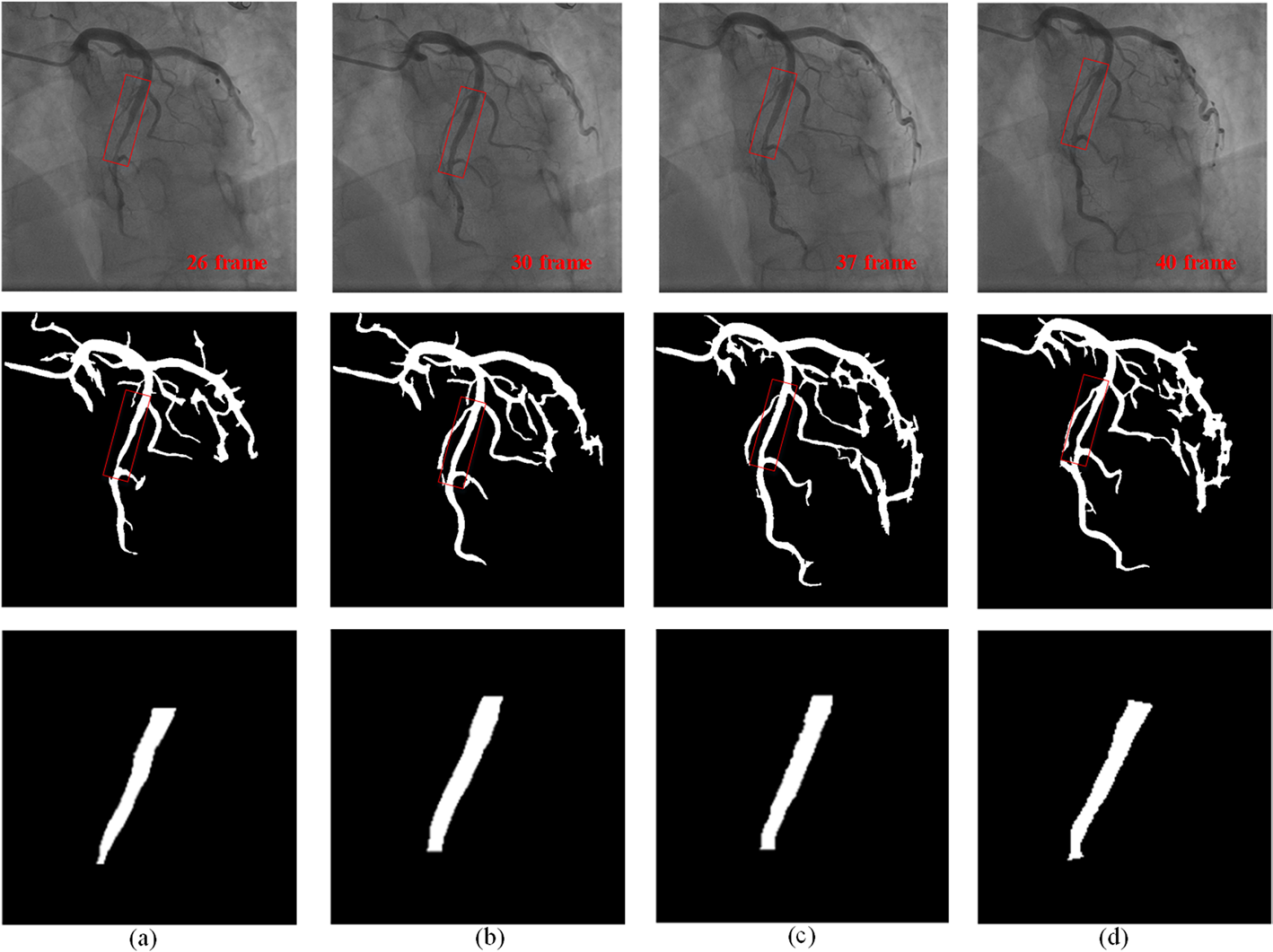
A case of myocardial bridge: The first row is the original image of 26, 30, 27, and 40 frames, respectively. The second row is the corresponding binary image; the third row is the diseased part

As a significant “milking effect” is present at the myocardial bridge on CCA, the whole process is shown on coronary angiography as periodic stenosis. Figure 8 shows a case of a myocardial bridge. Clinically, the physician diagnoses the myocardial bridge by observing the periodic stenosis of the vessels in the angiography sequence. Therefore, we propose an automatic detection method of the myocardial bridge that combines the detection of vessel stenosis and the fusion of coronary sequence information. We have observed that the myocardial bridge manifests as periodic stenosis on a single vessel or the entire vessel becomes thinner. Based on this feature, we diagnose the myocardial bridge by continuously querying the degree of stenosis and vessel diameter that match the vessel. Combined with the physician’s diagnostic myocardial bridge method on CCA and the actual situation of the vessel images, the diagnosis can be confirmed if one of the following conditions is met:

18 $$\left\{ {\begin{array}{*{20}c} {{\text{stenosis}}\_{\text{max}} - {\text{stenosis}}\_{\text{min}} \ge 0.25} \\ {{\text{stenosis}}\_{\text{max}}\_{\text{frame}} - {\text{stenosis}}\_{\text{min}}\_{\text{frame}} \ge 3} \\ \end{array} } \right.$$

19 $$\left\{ {\begin{array}{*{20}c} {{\text{average}}\_{\text{min}}/{\text{average}}\_{\text{max}} \le 0.75} \\ {{\text{average}}\_{\text{max}}\_{\text{frame}} - {\text{average}}\_{\text{min}}\_{\text{frame}} \ge 3} \\ \end{array} } \right.$$

In an angiographic sequence, we can calculate the extreme and average diameter of each vessel from the above. Where ${\text{stenosis}}\_{\text{max}}$ and ${\text{stenosis}}\_{\text{min}}$ denote the extreme value of the stenosis in the same vessel in the sequence in different images, respectively. ${\text{average}}\_{\text{max}}$ and ${\text{average}}\_{\text{min}}$, respectively, denote the extreme value of the average vessel diameter in the same vessel in different images. We refer to the definition of myocardial bridges and the grading of stenosis, and obtain the two thresholds through extensive experiments. To increase the robustness of detection, the spacing of the maximum and minimum values is at least greater than 3. Equations (15) and (16) are independent of each other. One of them is satisfied, it can be judged as myocardial bridge. Equation (16) is designed to accommodate cases where a long lesion makes it impossible to calculate the stenosis.

### Parameter settings

In coronary vessel segmentation, we performed experiments on pytorch with Intel Xeon(R) 6238R CPU and NVIDIA A40 GPU. The software environment contains python 3.8.12, pytorch 1.7 and CUDA 11.0. In experiments, we use a fivefold cross-validation approach with 150 epoch of training each time, dividing all the images into 80%, 2129 images, for the training set and 20%, 532 images, for the test set. We used the SGD optimizer and set the patch size to 8, the base learning rate to 0.05, and the momentum and weight decay to 0.9 and $1e - 4$, respectively.

We define vessels with a vessel length of less than 6 pixels as burr vessels, which is a comprehensive consideration of the diameter of the vessel and the skeleton algorithm. A large threshold may result in the removal of trunk vessels by mistake, while a small threshold may fail to achieve the deburring effect. When using the least-squares method to fit the straight line of the points on the vessel skeleton, we set $N$ to 5. $d_n$ is set as 10, which considers the impact of removing burr vessels to ensure that vessel tree is established. $d_s$ and $d_e$ are city block distance which is a way to represent distance in an image in image processing. The thresholds of $d_s$, $d_e$ and ${\text{rate}}$ are determined by the framerate, because the vessels appear to be displaced and folded between two adjacent images.

## Data Availability

Data and the programming code used as part of this research can be obtained from authors on a request.
